# High-throughput DNA barcoding of oligochaetes for abundance-based indices to assess the biological quality of sediments in streams and lakes

**DOI:** 10.1038/s41598-020-58703-2

**Published:** 2020-02-06

**Authors:** Régis Vivien, Laure Apothéloz-Perret-Gentil, Jan Pawlowski, Inge Werner, Michel Lafont, Benoit J. D. Ferrari

**Affiliations:** 1 Swiss Centre for Applied Ecotoxicology (Ecotox Centre), Lausanne/Dübendorf, Switzerland; 2 Department of Genetics and Evolution, University of Geneva, Geneva, Switzerland; 3 ID-Gene ecodiagnostics, Campus Biotech Innovation Park, 1202 Geneva, Switzerland; 4 Laboratoire d’Ecologie des Hydrosystèmes Naturels et Anthropisés, Université Lyon I, 69622 Villeurbanne, France; 5 Institute of Oceanology, Polish Academy of Sciences, Powstancow Warszawy 55, 81-712 Sopot, Poland

**Keywords:** Genetics, Molecular biology, Zoology, Ecology, Environmental sciences

## Abstract

Aquatic oligochaete communities are valuable indicators of the biological quality of sediments in streams and lakes, but identification of specimens to the species level based on morphological features requires solid expertise in taxonomy and is possible only for a fraction of specimens present in a sample. The identification of aquatic oligochaetes using DNA barcodes would facilitate their use in biomonitoring and allow a wider use of this taxonomic group for ecological diagnoses. Previous approaches based on DNA metabarcoding of samples composed of total sediments or pools of specimens have been proposed for assessing the biological quality of ecosystems, but such methods do not provide precise information on species abundance, which limits the value of resulting ecological diagnoses. Here, we tested how a DNA barcoding approach based on high-throughput sequencing of sorted and genetically tagged specimens performed to assess oligochaete species diversity and abundance and the biological quality of sediments in streams and lakes. We applied both molecular and morphological approaches at 13 sites in Swiss streams and at 7 sites in Lake Geneva. We genetically identified 33 or 66 specimens per site. For both approaches, we used the same index calculations. We found that the ecological diagnoses derived from the genetic approach matched well with those of the morphological approach and that the genetic identification of only 33 specimens per site provided enough ecological information for correctly estimating the biological quality of sediments in streams and lakes.

## Introduction

Aquatic oligochaetes are common in almost all freshwater ecosystems and comprise a large number of species with a wide range of pollution sensitivities^1^. Various biological methods based on the analysis of oligochaete assemblages have therefore been applied for assessing the quality of sediments in streams and lakes, among them the Oligochaete Index of Sediment Bioindication (IOBS)^2,3^ for streams and the Oligochaete Index of Lake Bioindication (IOBL)^2,4^. The morphological identification of oligochaetes to the species level is difficult, however, and only a part of the specimens present in a sample can usually be identified to the species level^5^. As a consequence, the difficulties associated with the identification of aquatic oligochaetes based on morphological features have prevented the more widespread use of this taxocenosis for ecological diagnostics.

The use DNA barcodes would greatly facilitate identification of aquatic oligochaete species and establishment of ecological diagnostics of sediments^5^. The mitochondrial cytochrome c oxidase (COI) barcode was suggested for identification of aquatic and terrestrial oligochaetes^6–8^. A 10% threshold of COI divergence has been considered appropriate for distinguishing aquatic oligochaete species^5,9–11^. However, this threshold needs adjustment for some species^12^. A reference database of COI sequences of aquatic oligochaetes based on the analysis of specimens collected in Switzerland is currently being developed^5^.

High-throughput sequencing allows the molecular analysis of a large number of samples at the same time and has been proposed as a cost-effective way to assess biodiversity in routine biomonitoring^13–15^. So far, all studies using this technology for assessing the biological quality of aquatic ecosystems have been based on the analysis of DNA extracted from water, sediments or a pool of specimens previously sorted ((e)DNA metabarcoding)^12^. The principal issue of such methodologies is the lack of precision in the abundance estimation of each species present in a sample^16–18^. This limits their application for ecological diagnoses as the calculations of biological indices are largely based on taxa abundances.

This abundance issue could be solved by sorting the specimens of a sample and tagging them genetically during PCR amplification before high-throughput sequencing. In the study presented here, we called this methodology “high-throughput DNA barcoding”. Shokralla *et al*.^19,20^ and Hebert *et al*.^21^ proposed this methodology as replacement of Sanger sequencing for purposes of molecular barcoding (establishment of databases of molecular barcodes) or biodiversity monitoring, but it could be also suitable for assessing the biological quality of an ecosystem. This approach is more time consuming than the (e)DNA metabarcoding methods, but it optimizes the quality of ecological diagnoses by providing correct and precise data on species abundance. Such a high-throughput DNA barcoding method would only be practicable for routine analyses if the number of specimens sequenced per site could be limited to less than 100. Identification of 100 oligochaete specimens is required for the morphological method^2^; however, only a part of the specimens can be identified morphologically to the species level.

In this study, we tested how the high-throughput DNA barcoding approach based on the analysis of sorted specimens compares to the conventional morphological approach for assessing oligochaete species diversity and abundance, and thus the biological quality of sediments in streams and lakes. We applied the two approaches at 13 sites in Swiss streams and at 7 sites in Lake Geneva. We genetically identified 33 (9 sites) or 66 (11 sites) specimens per site. Calculations of the molecular and morphological indices used the same formulas. We compared the ecological diagnoses established using both approaches and studied if the genetic identification of only 33 specimens per site was sufficient for correctly estimating the quality of sediments in streams and lakes.

## Material and Methods

### Sampling sites

Thirteen sites in streams of 3 cantons of Switzerland and 7 sites along the shores of Lake Geneva were selected to cover a known gradient of anthropogenic pressures, in order to compare the morphological and metabarcoding approaches for determining the oligochaete indices (Supplementary Table S1). The 13 stream sites comprised sources and sites in industrial, urban and agricultural areas. The 7 sites in Lake Geneva were selected based on a previous study on the physicochemical quality of sediments of this lake^22^ with sediments containing a range of metal concentrations.

### Sampling and sieving of samples for morphological and genetic analyses

Sediment samples (3 L) were collected and sieved according to IOBS and IOBL protocols^2^ using a Surber type net (0.2 mm mesh size) for stream sites and an Ekman type grab sampler for lake sites. The top 10 cm of sediments were collected in both stream and lake sites. The water depth where the samples were collected was 30–50 cm for stream sites and 20–70 m for lake sites. At each site (streams and lake), 3 subsamples (one sample every 10–20 meters) were collected, combined and fixed with 10% neutral buffered formalin (ThermoFisher Scientific, Ecublens, Switzerland) adjusted to a final formaldehyde concentration of 4%. Formalin optimally fixes oligochaete specimens, and a study showed that fixation and storage of oligochaete specimens in 4% neutral buffered formalin for up to 30 days was suitable for subsequent genetic analyses^23^. Sediment samples were stored at 4 °C for 1 to 5 days before sieving, then sieved through a column of sieves with 5 mm and 0.5 mm mesh size. The material retained on the 0.5 mm sieve was transferred to a plastic box and preserved in absolute ethanol at −20 °C. The big oligochaete specimens retained in the 5 mm sieve were incorporated in the plastic box.

### Morphological analysis

Morphological analyses of oligochaete samples followed IOBS and IOBL guidelines^2^. The preserved specimens were transferred to a subsampling square box (5 × 5 cells), and the contents of randomly selected cells were transferred into a Petri dish and examined under a stereomicroscope until 100 specimens were collected. In two samples from the sources of streams, oligochaete densities were very low and only 28 (source Boiron) and 43 specimens (L’isle) per site were obtained for morphological analysis. In these samples, the identification of low numbers of specimens had no impact on the results of ecological diagnoses as all specimens belonged to sensitive species indicating good sediment quality. Sorted specimens were then mounted on slides in a coating solution composed of lactic acid, glycerol and polyvinylic alcohol. Oligochaete specimens were identified to the lowest practical level (species if possible) using a compound microscope.

### Genetic analysis

#### Sorting of specimens

**Figure 1:**
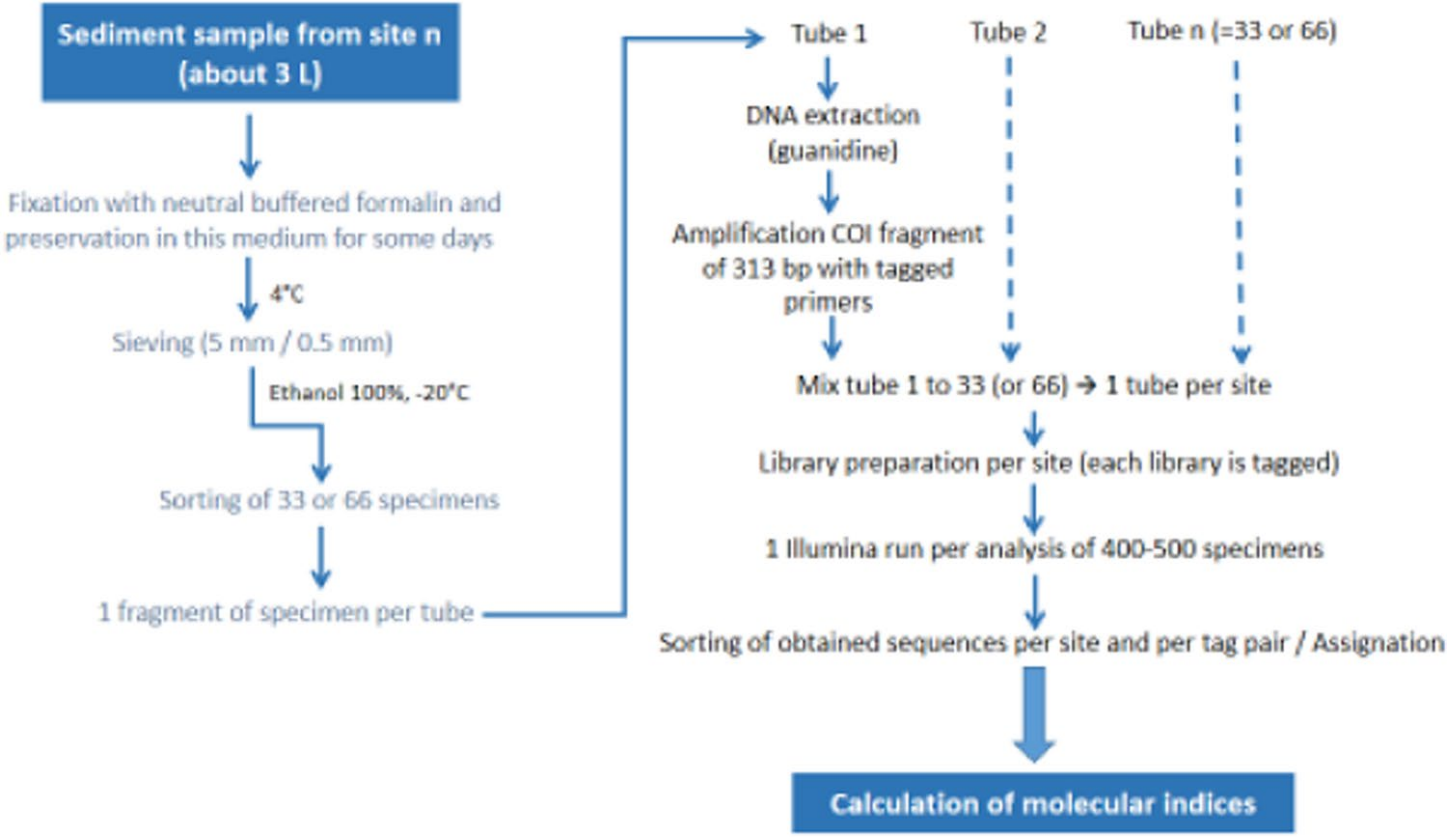
Workflow of the different steps of the high-throughput DNA barcoding analysis from fixation of oligochaete specimens to calculation of the indices.

As for morphological analysis, sieved material preserved in absolute ethanol at −20 °C was transferred into a subsampling square box (5 × 5 cells). The contents of randomly selected cells were examined under a stereomicroscope until 33 (9 sites) or 66 specimens (11 sites) were collected (Supplementary Table S1). We assumed that the identification of 33 specimens (corresponding to about 1/3 of the number of morphologically identified specimens) to the species level per site could be enough for assessing the biological quality of sediments. For the 11 sites where 66 specimens were collected, we sorted out two sets of 33 specimens to examine the differences obtained in species diversity and abundance by analysing 33 or 66 specimens, and to determine if the analysis of only 33 specimens was sufficient to establish correct ecological diagnoses. The posterior region of each specimen was cut off transversally and transferred to a 1 ml tube containing 0.03 ml absolute ethanol (1 tube per specimen). The anterior part of some specimens (12 to 24 per site) was preserved in absolute ethanol for further morphological identification, in case some of the preserved specimens corresponded to new lineages (=species), i.e. were unassigned using our Swiss COI reference database^5^ or GenBank public database (NCBI). Specimen samples for genetic analysis were stored at −20 °C until DNA extraction. Before DNA extraction, samples were dried for 24 hours at room temperature. The workflow from fixation of specimens to calculation of genetic indices is shown in Fig. 1.

#### DNA extraction, PCR amplification, library preparation and Illumina sequencing

Total genomic DNA was extracted from tissue samples using the guanidine thiocyanate method described by Tkach and Pawlowski (1999)^24^. The primers specific to metazoans “mlCOIintF” and “jgHCO2198”^25^ were used to amplify a COI fragment (313 base pairs) from each DNA extract. PCR amplifications were performed in a total volume of 50 μl containing 0.5 μl of Taq polymerase 5U/μl (Roche, Basel, Switzerland), 5 μl of the PCR buffer (10x concentrated) with MgCl_2_ (Roche), 1.25 μl of each primer (10 μM each), 1 μl of a mix containing 10 mM of each dNTP (Roche) and 2.5 μl of DNA template. The PCR comprised an initial denaturation step at 95 °C for 5 min, followed by 35 cycles of denaturation at 95 °C for 40 s, annealing at 44 °C for 45 s and elongation at 72 °C for 1 min and a final elongation step at 72 °C for 8 min.

The metazoan primers were tagged by bearing 8 nucleotides attached at each primer’s 5′ extremity. A unique combination of tagged primers was used for each specimen in order to multiplex all specimens in a unique sequencing library^26^. PCR products of each specimen were quantified with capillary electrophoresis using QIAxcel instrument (Qiagen, Hilden, Germany). Equimolar concentrations of PCR products were pooled into a single tube (one tube per site) and purified using High Pure PCR Product Purification kit (Roche Diagnostics). Then, 700 ng of purified PCR products was appended with Illumina PE adapter sequences in order to obtain one functional sequencing library per PCR sample (or site). This was performed using the TruSeq DNA PCR-Free Library Preparation Kit (Illumina) following the kit instructions to include a unique index as a label for each library. The libraries were quantified by qPCR using the KAPA Library Quantification Kit (Roche). Finally, libraries were sequenced on a MiSeq instrument using paired-end sequencing for 500 cycles with nano kit v2.

The raw sequences are accessible in the Short Read Archive under the BioProject number PRJNA563268. The COI sequences of 313 bp corresponding to new lineages for our local reference COI database (one sequence per lineage) obtained as part of this study are provided in Supplementary File S1. In the near future, we will Sanger sequence a large segment of COI (658 bp) using universal primers^27^ of the specimens corresponding to these lineages. We will deposit these sequences (658 bp) in the European Nucleotide Archive as part of a future publication on the update of our COI reference database.

#### Analysis of sequences

Bioinformatic analyses were performed using an in-house pipeline (SLIM^28^). Raw fastq reads were quality-filtered by removing any sequence with a mean quality score of 30 and removing all sequences with ambiguous bases or any mismatch in the tagged primer. Paired-end reads were then assembled using simple bayesian algorithm implemented in PANDAseq^29^. Chimera removing and the OTUs clustering at 97% were performed using VSEARCH^30^.

To assign the sequence corresponding to each tagged specimen to a specific lineage (or species), we considered that the sequences diverging by less than 10% (in COI) belonged to the same species, except for some species within the genera *Nais* and *Uncinais* (8%)^5^. The obtained sequences were first taxonomically assigned based on our local COI reference database^5^ using VSEARCH algorithms^30^. Then, each unassigned sequence using our local reference database was taxonomically assigned based on GenBank database using BLAST (http://www.ncbi.nlm.nih.gov/BLAST/Blast.cgi). Sequences that could not be assigned using these databases were identified either through morphological analysis of corresponding anterior part or by building barcode trees. Specimens taxonomically assigned using such trees were identified to the family or subfamily level. To construct these trees, the neighbour-joining method as implemented in Seaview v.4.4.0 was applied^31^, with 1,000 bootstrap replicates. The genetic distances between our sequences and GenBank’s sequences and between the sequences taxonomically assigned by building barcode trees were calculated using the K2P model in MEGA 5.1^32^.

### Oligochaete indices

To analyse the genetic data, we applied the same index calculations (for streams and lake) as for the morphological analysis:

For assessing the biological quality of fine/sandy sediments of streams we applied the IOBS index^2,3^ calculated according to the following formula:

$${\rm{IOBS}}=10{{\rm{ST}}}^{-1}$$

where S is the total number of taxa identified among 100 oligochaete specimens examined per sample, and T is the percentage of tubificids with or without hair setae that is dominant in the sediment sample (mature and immature worms combined). The index ranks the biological quality of sediments as follows: IOBS ≥ 6: very good, 3–5.9: good, 2–2.9: medium, 1–1.9: poor, <1: bad.

The biological quality of lake sediments was assessed using the “percentage of sensitive taxa” to pollution, as described in the IOBL guideline, which also contains a list of sensitive oligochaete taxa in lakes^2,4^. To this list, we added the species *Spirosperma ferox*, considered in lakes as sensitive by Lods-Crozet & Reymond (2005)^33^. The biological quality is ranked as follows: percentage >50: very good, 21–50: good, 11–20: medium, 6–10: poor, 0–5: bad. The IOBL index itself was not calculated. This index assesses the functioning of the lake sediments (ranks from low to high metabolic potential) and takes into account the total number of taxa and oligochaete density^4^. It is therefore not suitable for a comparison between morphological and genetic results as the two approaches would be compared only based on the total number of taxa.

### Statistical analyses

Linear regressions between the IOBS index values, percentages of sensitive taxa (lake) and percentages of the families/subfamilies that were frequent in our samples (Tubificinae with hair setae, Tubificinae without hair setae, Naidinae, Pristininae, Lumbriculidae and Enchytraeidae) obtained using the morphological and genetic data were performed. For each relationship, we determined the coefficient of determination R^2^, the slope (a) of the linear regression line genetic (y)/morphology (x) (y = ax + b) and applied the Pearson test. These analyses were performed using the Free Statistics and Forecasting Software^34^. Prior to statistical analysis, a log-linearization was applied to the IOBS data.

## Results

### Oligochaete diversity

In streams, about 1170 specimens were identified morphologically versus 693 genetically. In Lake Geneva, 700 specimens were identified morphologically versus 330 genetically. Almost all specimens (>99%) were successfully sequenced and assigned to taxa. In stream samples, 38 taxa were identified morphologically (16 Tubificinae, 6 Naidinae, 1 Pristininae, 4 Lumbriculidae, 9 Enchytraeidae and 2 Lumbricidae), while 63 lineages were identified genetically (30 Tubificinae, 7 Naidinae, 1 Prisininae, 3 Lumbriculidae, 20 Enchytraeidae and 2 Rhyacodrilinae) (Supplementary Tables S2 and S3). Of these 63 lineages, 51 were identified using existing databases (45 with the Swiss database, 6 with GenBank), 4 by morphological analysis and 8 by constructing a barcode tree. In the lake, 24 taxa were identified morphologically (17 Tubificinae, 4 Naidinae and 3 Lumbriculidae), while 30 lineages were identified genetically (23 Tubificinae, 4 Naidinae, 2 Lumbriculidae and 1 Haplotaxidae) (Supplementary Tables S4 and S5). Of these 30 lineages, 24 were identified using the Swiss database, 5 by morphological analysis and 1 by constructing a barcode tree. Eighteen new lineages (i.e. unassigned in the Swiss database and GenBank) were found in stream and lake sediments. Most of these (11) were identified to the family/subfamily or genus level.

While only about half the number of specimens were analysed genetically, more taxa were identified overall by the high-throughput DNA barcoding approach than by morphological analysis. This can be explained by the genetic detection of species which are very difficult or impossible to identify morphologically. For example, we were able to identify several cryptic species in *Tubifex tubifex* and *Limnodrilus hoffmeisteri* and some species in the family Enchytraeidae (*Fridericia perrieri*, *Buchholzia appendiculata*, etc.) identifiable morphologically only when the specimens are in a mature state and by using dissection. In addition, several specimens morphologically identified as Enchytraeidae g. sp. were found to be several distinct lineages.

Identification of only 33 specimens per sample proved sufficient for obtaining good agreement with morphological analysis (based on 100 specimens) and genetic analysis obtained with 66 specimens. While the number of taxa per site obtained by morphological analysis was higher in 11 of 20 samples than the number of lineages obtained by sequencing 33 specimens (Supplementary Table S6), it was identical for 3 samples and lower for 6 samples. When we sequenced 66 specimens per site, the number of lineages identified was higher than the number of taxa identified morphologically for 6 of 11 samples, identical for 2 samples and lower for 3 samples. The correlations between the percentages of Tubificinae, Tubificinae with hair setae, Tubificinae without hair setae, Naidinae, Pristininae, Lumbriculidae and Enchytraeidae obtained with the morphological analysis and the genetic identification of 33 or 66 specimens were highly significant (Table 1, Supplementary Table S6).

**Table 1:** R^2^, p value and slope (of the linear regression line y = ax + b) of the relationships between the percentages of families/subfamilies obtained using the morphological (x) and high-throughput DNA barcoding of 33 and 66 specimens (y) analyses (results from the stream and lake sites combined).

		% Tubificinae	% Tubificinae without hair setae	% Tubificinae with hair setae	% Naidinae + Pristininae	% Lumbriculidae	% Enchytraeidae
33 specimens, n = 20	R^2^	0.980	0.937	0.927	0.956	0.819	0.987
	p	1.1*10^−16^	2.9*10^−12^	1.2*10^−11^	1.2*10^−13^	4*10^−8^	1.9*10^−18^
	slope	0.954	0.962	1.012	0.834	1.137	1.038
66 specimens, n = 11	R^2^	0.998	0.962	0.974	0.994	0.888	0.895
	p	2.4*10^−13^	9.9*10^−8^	2.0*10^−8^	3.6*10^−11^	1.4*10^−5^	1.2*10^−5^
	slope	0.990	0.976	1.007	0.929	0.907	2.853

### Comparison of oligochaete indices derived from morphological and genetic data

Resulting IOBS indices for streams sites calculated with morphological data of 100 specimens and genetic data for 33 (n = 13; R^2^ = 0.851; p = 7.2*10^−6^; slope = 0.993) or 66 (n = 8; R^2^ = 0.935; p = 8.8*10^−5^; slope = 0.813) specimens were generally concordant (Tables 2 and 3), and correlations were highly significant. Similarly, the percentage of sensitive taxa at lake sites determined morphologically was highly correlated with both the percentage of sensitive taxa based on genetic analysis of 33 specimens (n = 7; R^2^ = 0.954; p = 0.0002; slope = 1.009) and 66 specimens (n = 3; R^2^ = 0.991; p value not calculated; slope = 1.114).

**Table 2:**
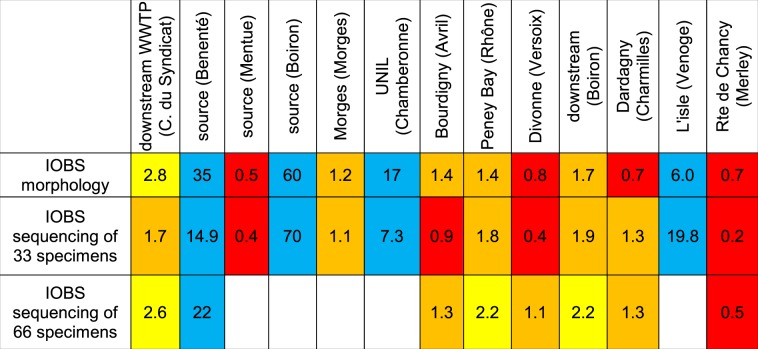
IOBS values obtained with morphological analysis and high-throughput DNA barcoding of 33 and 66 specimens.

**Table 3:**
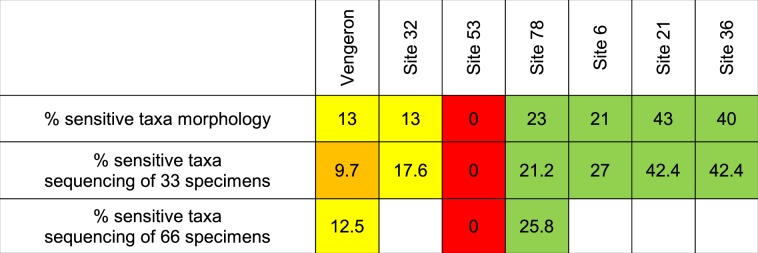
Percentage of sensitive species at sampling sites in Lake Geneva obtained with morphological analysis and high-throughput DNA barcoding of 33 and 66 specimens.

The classification of biological quality of sediments resulting from 33 specimens identified by high-throughput DNA barcoding was identical to results of the morphological analysis for 16 out of 20 samples (results from stream and lake sites combined), slightly higher for one sample and slightly lower for 3 samples (quality bad-poor at one site and quality poor-medium at two sites) (Tables 2 and 3). In 2 of these 3 samples, the number of taxa/species obtained by genetic analysis was lower than by morphological analysis, while in the third (a lake sample), the percentage of some sensitive species obtained genetically was underestimated. The differences disappeared when 33 additional specimens from each sample were identified genetically, due to an increase of the number of taxa identified and/or higher precision in the quantification of species abundances.

Sediment quality categories obtained by sequencing 66 specimens were identical to results of the morphological analysis for 7 of 11 samples (results from stream and lake sites combined) and slightly better for the four remaining samples (quality poor-medium and bad-poor). In these 4 samples, numbers of genetically identified species/taxa were higher than those identified morphologically.

## Discussion

Our study showed that high-throughput DNA barcoding of single sorted specimens was suitable to assess oligochaete diversity and the biological quality of sediments. The results of the morphological and high-throughput DNA barcoding approaches agreed well with each other. Only a few disagreements were observed within the “good quality not achieved” class. To our knowledge, it is the first time that this approach was tested for assessing the biological quality of ecosystems.

Even if the genetic identification of 66 specimens per site resulted in a higher richness of lineages than the genetic identification of 33 specimens, the classification of sites derived from the analysis of these two different numbers of specimens were very similar. There were some differences in the number of taxa identified by genetic and morphological analyses, but the accurate estimation of tubificids with and without hair setae obtained using the genetic approach minimized the impact of these differences on the categorization into quality classes. The genetic identification of more than 66 specimens seems therefore not necessary for establishing ecological diagnoses and can result in an increase of the number of detected taxa, which could imply to adapt the calculation of the biological index to avoid discordances between the genetic and morphological analyses (the IOBS index calculation takes into account the total number of taxa). Conversely, the identification of less than 33 specimens can in some cases lead to an underestimation of sediment quality due to the detection of lower numbers of taxa and to some imprecision in species abundances.

The time- and cost-effectiveness of high-throughput DNA barcoding of single sorted specimens strongly depends on the number of analyzed specimens per site. It is evident that this approach would be applicable in routine biomonitoring only if a restricted number of specimens was analyzed per site. A compromise should therefore be found between the applicability of this approach in routine (reasonable time and costs) and the imperative to ensure correct quality of the ecological diagnoses. In view of our study, the analysis of 33 specimens per site provides reliable assessments of sediment quality while being not too time consuming.

It is important to continuously enrich the COI reference database in parallel to the development of the genetic indices to optimize the quality of ecological diagnoses. The local database and oligochaete inventories can be efficiently complemented by preserving the anterior parts of the analysed specimens and by morphologically identifying them once the specimens have been sequenced. In this study, out of a total of 18 new lineages detected, 9 were identified morphologically, among them 7 to the species level. It is essential that the COI database contains sequences identified to the species level, if possible. Indeed, the ecology of lineages assigned to the family or subfamily level is often uncertain. For example, even if most taxa in Enchytraeidae and Lumbriculidae are classified as sensitive to pollution, these families include some resistant and polyphyletic taxa, for example *Enchytraeus buchholzi* (Enchytraeidae) and *Lumbriculus variegatus* (Lumbriculidae)^5,35^. In addition, new lineages of tubificids cannot be easily assigned to the groups with or without hair setae as these two groups are not monophyletic^5^. In our dataset, most of the new lineages were identified to the subfamily or family level, but this had no impact on the results of environmental diagnoses as these new lineages were mainly found in streams and within other families/subfamilies than tubificids (the IOBS index calculation takes into account the percentage of tubificids with or without hair setae).

In conclusion, we have shown that the high-throughput DNA barcoding of single tagged oligochaete specimens represents a sound alternative to the classical morphological approach for assessing the biological quality of sediments in streams and lakes. The molecular identification of 33 oligochaete specimens proved sufficient for correctly estimating the oligochaete metrics and therefore the quality of sediments. However, we suggest to sequence about 45–50 specimens per site, if this number is not too high for routine analyses, to optimize the detection of species and the estimation of species abundance. This new method has excellent potential to replace the morphological method and allow a wide use of oligochaetes as bioindicators for sediment quality, especially because the molecular approach can be applied by non-experts in the systematics of this organism group. To achieve this goal it is important to complement the COI reference database, and to increase the applicability of this approach in routine analyses by facilitating and shortening the different steps of the analysis, in particular DNA extraction and PCR amplification.

## Supplementary Information

Supplementary Information.

## Data Availability

All data generated or analyzed during this study are included in the manuscript and its Supplementary Information files. Raw sequences are accessible in the Short Read Archive under the BioProject number PRJNA563268. The COI sequences of 313 bp corresponding to new lineages for our local reference COI database (one sequence per lineage) obtained as part of this study are provided in Supplementary File S1. In the near future, we will Sanger sequence a large segment of COI (658 bp) using the universal primers (HCO and LCO) of the specimens corresponding to these lineages. We will deposit these sequences (658 bp) in the European Nucleotide Archive as part of a future publication on the update of our COI reference database.
