# Supplementary material for: High-throughput DNA barcoding of oligochaetes for abundance-based indices to assess the biological quality of sediments in streams and lakes
Source: Sci Rep. 2020 Feb 6;10:2041. doi: 10.1038/s41598-020-58703-2 (PMC7005023; doi:10.1038/s41598-020-58703-2)
Supplement: Supplementary file 1 — Supplementary Information. [file 41598_2020_58703_MOESM1_ESM.pdf]

High-throughput DNA barcoding of oligochaetes for abundance-based indices to assess the biological quality of sediments in streams and lakes

Régis Vivien[1*], Laure Apothéloz-Perret-Gentil[2, 3], Jan Pawlowski[2, 3, 5], Inge Werner[1], Michel Lafont[4], Benoit J.D. Ferrari[1]

[1] Swiss Centre for Applied Ecotoxicology (Ecotox Centre), Lausanne/Dübendorf, Switzerland
[2] Department of Genetics and Evolution, University of Geneva, Geneva, Switzerland
[3] ID-Gene ecodiagnostics, Campus Biotech Innovation Park, 1202 Geneva, Switzerland
[4] Laboratoire d'Ecologie des Hydrosystèmes Naturels et Anthropisés, Université Lyon I, 69622 Villeurbanne, France
[5] Institute of Oceanology, Polish Academy of Sciences, Powstancow Warszawy 55, 81-712 Sopot, Poland

[*] Corresponding author: regis.vivien@centreecotox.ch

site	river / lake	Sampling date	X Y coordinates	Performed analyses		
				Morphology	High-throughput DNA barcoding 33 specimens	High-throughput DNA barcoding 66 specimens
downstream WWTP	Canal du Syndicat	27/03/2017	46°08'23.454'' N 7°09'10.704'' E	x	x	x
source	Benenté	29/9/2017	46°35'13.250'' N 6°39'54.654'' E	x	x	x
source	Mentue	29/9/2017	46°36'00.527'' N 6°42'50.866'' E	x	x	
source	Boiron	16/10/2017	46°31'49.890'' N 6°22'59.182'' E	x	x	
Morges	Morges	26/02/2018	46°30'47.148'' N 6°29'15.369'' E	x	x	
UNIL	Chamberonne	09/03/2018	46°31'22.261'' N 6°34'57.729'' E	x	x	
Bourdigny	Avril	06/02/2018	46°13'01.028'' N 6°02'48.770'' E	x	x	x
Peney Bay	Rhône	06/02/2018	46°12'03.374'' N 6°02'35.551'' E	x	x	x
Divonne	Versoix	17/05/2018	46°20′24.243″ N 6°08′25.635″ E	x	x	x
downstream	Boiron	16/10/2018	46°29′36.335″ N 6°27′10.915″ E	x	x	x
Dardagny	Charmilles	18/09/2018	46°11′21.664″ N 5°59′37.888″ E	x	x	x
L'isle	Venoge	04/01/2019	46°37′05.56″ N 6°24′26.60″ E	x	x	
Rte de chancy	Merley	18/09/18	46°10′22.052″ N 6°02′36.751″ E	x	x	x
Site 32	Lake Geneva	26/10/2017	46°27'58.242'' N 6°25'23.376'' E	x	x	
Site 53 (Vidy Bay)	Lake Geneva	26/10/2017	46°30'36.508'' N 6°35'17.172'' E	x	x	x
Site 78	Lake Geneva	26/10/2017	46°24'35.945'' N 6°53'33.417'' E	x	x	x
Vengeron	Lake Geneva	04/09/17	46°14′43.360″ N 6°09′27.276″ E	x	x	x
Site 6	Lake Geneva	22/05/2018	46°17'47.059'' N 6°11'33.744'' E	x	x	
Site 21	Lake Geneva	22/05/2018	46°22'47.461'' N 6°19'52.258'' E	x	x	
Site 36	Lake Geneva	22/05/2018	46°22'15.661'' N 6°27'03.507'' E	x	x	

Supplementary Table S1: Sampling details and performed analyses per site (x=analysis performed)

		downstream WWTP (Canal du Syndicat)	source (Benenté)	source (Mentue)	source (Boiron)	Morges (Morges)	UNIL (Chamberonne)	Bourdigny (Avril)	Peney Bay (Avril)	Divonne (Versoix)	downstream (Boiron)	Dardagny (Charmilles)	L'isle (Venoge)	Rte de Chancy (Merley)
Tubificinae	Tubificinae with hair setae (unidentifiable)	19	2	78		1	3	30	17	68	27	26		90
	Tubifex tubifex			5				3	1	5	1			1
	Lophochaeta ignota										7			
	Aulodrilus pluriseta						1							
	Psammoryctides barbatus						1							
	Potamothrix bavaricus							11	1					
	Potamothrix hammoniensis								8					
	Tubificinae without hair setae (unidentifiable)	18				48	6	47	57	10	52	64		5
	Limnodrilus hoffmeisteri	11				16	3	5	4	5		1		1
	Limnodrilus claparedianus	2				1		2	1		2			1
	Limnodrilus profundicola	1				2		1						
	Limnodrilus udekemianus						1		3		2	10		
	Aulodrilus limnobius		3											
	Potamothrix moldaviensis					22								
Rhyacodrilinae	*Rhyacodrilus falciformis*												2	
	Rhyacodrilus sp.												3	
Pristininae	*Pristina jenkinae*		84			1								
Naidinae	*Nais communis*	2	3				1			1				
	Nais elinguis	43					44			9				
	Nais christinae	1					1							
	Nais barbata	2												
	Dero digitata											2		

		downstream WWTP (Canal du Syndicat)	source (Benenté)	source (Mentue)	source (Boiron)	Morges (Morges)	UNIL (Chamberonne)	Bourdigny (Avril)	Peney Bay (Avril)	Divonne (Versoix)	downstream (Boiron)	Dardagny (Charmilles)	L'isle (Venoge)	Rte de Chancy (Merley)
	Vejdovskyella intermedia					2								
Lumbriculidae	Lumbriculidae g. sp. (unidentifiable)		4	1	3	2	17				8			2
	Stylodrilus heringianus						8				4			
	Lumbriculus variegatus							1	10					
	Dorydrilus michaelseni or *Stylodrilus lemani*				1									
Enchytraeidae	*Lumbricillus* sp.						1							
	Enchytraeus buchholzi						3						4	
	Cernosvitoviella sp.			16	3									
	Achaeta sp.												10	
	Fridericia sp.		2		6		4						7	
	Henlea sp.				8		1							
	Cognettia sp.				3								3	
	Marionina argentea		2		4						1		14	
	Globulidrilus riparius						6							
Lumbricidae	Lumbricidae g. sp.					1								
	Eiseniella tetraedra					1	1							

Supplementary Table S2: Percentages of oligochaete taxa in stream sites obtained with the morphological analysis

		downstream WWTP (C. du Syndicat)	downstream WWTP (C. du Syndicat)	source (Benenté)	source (Benenté)	source (Mentue)	source (Boiron)	Morges (Morges)	UNIL (Chamberonne)	Bourdigny (Avril)	Bourdigny (Avril)	Peney Bay (Rhône)	Peney Bay (Rhône)	Divonne (Versoix)	Divonne (Versoix)	downstream (Boiron)	downstream (Boiron)	Dardagny (Charmilles)	Dardagny (Charmilles)	L'isle (Venoge)	Rte de Chancy (Merley)	Rte de Chancy (Merley)
		33 s	66 s	33 s	66 s	33 s	33 s	33 s	33 s	33 s	66 s	33 s	66 s	33 s	66 s	33 s	66 s	33 s	66 s	33 s	33 s	66 s
Tubificinae	T1_Tubificinae sp. *											1	4									
	T2_Tubificinae sp. *	4	9						1							4	10					
	T3_Tubificinae sp. *												1									
	T4_*Aulodrilus pluriseta* *								1			1	1									
	T6_*Lophochaeta ignota* *															4	8					
	T7_*Potamothrix bavaricus* *									12	20											
	T9_*Tubifex tubifex* *			1	1						2	1	1		1			1	1			
	T10_*Tubifex tubifex* *																	1	3		31	56
	T11_*Tubifex tubifex* *		1	1	2	24				1	2			24	44	3	7	1	3			5
	T12_*Tubifex tubifex* *											1	1		1			4	7			
	T24_*Spirosperma ferox* *																					
	T26_*Tubifex* sp. *																					
	T27_*Tubifex tubifex* *														1			2	5			
	T28_*Potamothrix hammoniensis* *											4	10									
	T29_*Potamothrix vejdovskyi* *																					
	T31_*Potamothrix heuscheri* *																					
	T32_Tubificinae sp. *											1	2									
	Tubificinae sp. (with hair setae, *Tubifex tubifex* ?) ***																	1	1			
	T15_Tubificinae sp. (without hair setae) *									18	34	9	18									

		downstream WWTP (C. du Syndicat)	downstream WWTP (C. du Syndicat)	source (Benenté)	source (Benenté)	source (Mentue)	source (Boiron)	Morges (Morges)	UNIL (Chamberonne)	Bourdigny (Avril)	Bourdigny (Avril)	Peney Bay (Rhône)	Peney Bay (Rhône)	Divonne (Versoix)	Divonne (Versoix)	downstream (Boiron)	downstream (Boiron)	Dardagny (Charmilles)	Dardagny (Charmilles)	L'isle (Venoge)	Rte de Chancy (Merley)	Rte de Chancy (Merley)
		33 s	66 s	33 s	66 s	33 s	33 s	33 s	33 s	33 s	66 s	33 s	66 s	33 s	66 s	33 s	66 s	33 s	66 s	33 s	33 s	66 s
	T16_*Limnodrilus hoffmeisteri* *												1									
	T17_*Limnodrilus hoffmeisteri* *	8	15					7	5		5	2	3	4	9	7	11	2	3		2	4
	T18_*Limnodrilus hoffmeisteri* *	1	3									7	15		1	7	16	14	31			
	T19_*Limnodrilus hoffmeisteri* *							7														
	T20_*Limnodrilus hoffmeisteri* *	3	5												1							
	T22_*Limnodrilus claparedianus* *							2				2	2				3					1
	T23_*Limnodrilus udekemianus* *															1	1					
	T30_*Potamothrix moldaviensis* *							11														
	Limnodrilus claparedianus cervix **									1	1											
	Limnodrilus udekemianus ****							1								2	2	7	12			
	Aulodrilus limnobius ****			2	2																	
Lumbriculidae	LL3_*Stylodrilus heringianus* *			1	1	1	4	2	9							3	6					
	LL2_*Lumbriculus variegatus* *								1	1	1	4	7									
	Lumbriculidae sp. ****						1															
Naidinae	N11_*Nais christinae* *		1																			
	N3_*Nais communis* *			1	1																	
	N10_*Nais communis* *								1													
	N4_*Nais elinguis* *	16	31					1	11					3	6							
	N6_*Piguetiella blanci* *																					
	N7_*Vejdovskyella intermedia* *							1								1	1					

		downstream WWTP (C. du Syndicat)	downstream WWTP (C. du Syndicat)	source (Benenté)	source (Benenté)	source (Mentue)	source (Boiron)	Morges (Morges)	UNIL (Chamberonne)	Bourdigny (Avril)	Bourdigny (Avril)	Peney Bay (Rhône)	Peney Bay (Rhône)	Divonne (Versoix)	Divonne (Versoix)	downstream (Boiron)	downstream (Boiron)	Dardagny (Charmilles)	Dardagny (Charmilles)	L'isle (Venoge)	Rte de Chancy (Merley)	Rte de Chancy (Merley)
		33 s	66 s	33 s	66 s	33 s	33 s	33 s	33 s	33 s	66 s	33 s	66 s	33 s	66 s	33 s	66 s	33 s	66 s	33 s	33 s	66 s
	N9_*Nais communis* *	1	1																			
	P1_*Pristina jenkinae* *			22	51											1	1					
Rhyacodrilinae	*Rhyacodrilus falciformis* ** / ****																			2		
	R1_*Bothrioneurum vejdovskyanum* *										1											
Enchytraeidae	E16_*Henlea perpusilla* *						8													3		
	E4_*Marionina argentea* *							1														
	E5_*Marionina argentea* *			3	3		9													1		
	Fridericia perrieri **			1	2																	
	Enchytraeidae sp. (1) ***			1	1																	
	Enchytraeidae sp. (3) ***						1													1		
	Enchytraeidae sp. (4) ***				2																	
	Cernosvitoviella sp. ****					8	4															
	Chamaedrilus chalupskyi **						5													2		
	E11_*Globulidrilus riparius* *								1													
	E17_*Enchytraeus buchholzi* *								1													
	Buchholzia appendiculata **								1													
	Henlea ventriculosa **								1													
	E13_*Lumbricillus* sp. *																			2		
	E6_*Achaeta* sp. *																			10		
	E7_*Achaeta* sp. *																			5		

		downstream WWTP (C. du Syndicat)	downstream WWTP (C. du Syndicat)	source (Benenté)	source (Benenté)	source (Mentue)	source (Boiron)	Morges (Morges)	UNIL (Chamberonne)	Bourdigny (Avril)	Bourdigny (Avril)	Peney Bay (Rhône)	Peney Bay (Rhône)	Divonne (Versoix)	Divonne (Versoix)	downstream (Boiron)	downstream (Boiron)	Dardagny (Charmilles)	Dardagny (Charmilles)	L'isle (Venoge)	Rte de Chancy (Merley)	Rte de Chancy (Merley)
		33 s	66 s	33 s	66 s	33 s	33 s	33 s	33 s	33 s	66 s	33 s	66 s	33 s	66 s	33 s	66 s	33 s	66 s	33 s	33 s	66 s
	Enchytraeidae sp. (6, 27) ***																			2		
	Enchytraeidae sp. (17, 25) ***																			2		
	Enchytraeidae sp. (20, 21) ***																			2		
	Enchytraeidae sp. (32) ***																			1		

Supplementary Table S3: number of specimens of each oligochaete species/lineage obtained per stream site with high-throughput DNA barcoding of 33 (33 s) and 66 specimens (66 s); * taxa identified using our local COI reference database; ** taxa identified using Genbank data; *** taxa identified by building a barcode tree; **** taxa identified by morphological analysis

		Vengeron	Site 32	Site 53	Site 78	Site 6	Site 36	Site 21
Tubificinae	Tubificinae with hair setae (unidentifiable)	33	18	50	17	36	18	6
	Tubifex tubifex			6	1	2	4	
	Aulodrilus pluriseta		12		2	1	2	
	Psammoryctides barbatus (1)	2			16	10	2	
	Psammoryctides moravicus	3						
	Branchiura sowerbyi							
	Embolocephalus velutinus (1)						18	24
	Spirosperma ferox (1)	5				6	6	4
	Potamothrix heuscheri	1	1		1	3		
	Potamothrix hammoniensis				1	3		
	Potamothrix vejdovskyi	1	5		29		2	1
	Quistadrilus multisetosus			13				
	Potamothrix bavaricus							
	Tubifex ignotus							
	Tubificinae without hair setae (unidentifiable)	25	39	22	18	29	28	25
	Limnodrilus hoffmeisteri	11	2	11	2	4	1	3
	Limnodrilus claparedianus				1	1	2	
	Limnodrilus profundicola		1	1	2	3		1
	Aulodrilus limnobius	3	6					
	Potamothrix moldaviensis	7					6	5
Lumbriculidae	Lumbriculidae g. sp. (unidentifiable) (1)	1				4	10	3
	Dorydrilus michaelseni (1)							
	Stylodrilus heringianus (1)							
	Stylodrilus lemani (1)					1		
	Lumbriculus variegatus	2			1			
Naidinae	*Ophidonais serpentina* (1)							
	Piguetiella blanci (1)	5	3		1		2	
	Specaria josinae (1)							
	Uncinais uncinata (1)							
	Vejdovskiella intermedia (1)				6			

		Vengeron	Site 32	Site 53	Site 78	Site 6	Site 36	Site 21
	Stylaria lacustris (1)		10				2	
	Nais pardalis (1)							
	Dero digitata	1	2					

Supplementary Table S4: Percentages of oligochaete taxa in lake sites obtained with the morphological analysis. The names of taxa followed by (1) correspond to sensitive taxa

		Vengeron	Vengeron	Site 32	Site 53	Site 53	Site 78	Site 78	Site 6	Site 21	Site 36
		33 s	66 s	33 s	33 s	66 s	33 s	66 s	33 s	33 s	33 s
Tubificinae	T2_Tubificinae sp. *	6	11	3			5	7		4	2
	T4_*Aulodrilus pluriseta* *			5							
	T8_*Psammoryctides barbatus* (1) *		1				4	12	2		
	T9_*Tubifex tubifex* *				1	3				1	
	T11_*Tubifex tubifex* *				21	35			1	1	2
	T12_*Tubifex tubifex* *					1			1		
	T24_*Spirosperma ferox* (1) *	2	3						2	3	2
	T25_*Embolocephalus velutinus* (1) *									10	5
	T26_*Tubifex* sp. *	4	7	2							
	T27_*Tubifex tubifex* *									2	
	T28_*Potamothrix hammoniensis* *					1	2	2	7		
	T29_*Potamothrix vejdovskyi* *			2			11	24			
	T31_*Potamothrix heuscheri* *	4	5				2	2	3	2	1
	Tubificinae sp. (with hair setae) ***						1	1			
	Quistadrilus multisetosus ****				5	9					
	T15_Tubificinae sp. (without hair setae) *	2	6	3		2					
	T17_*Limnodrilus hoffmeisteri* *	2	4		4	11			3		1
	T18_*Limnodrilus hoffmeisteri* *	1	4	9						1	
	T19_*Limnodrilus hoffmeisteri* *	1	1								
	T20_*Limnodrilus hoffmeisteri* *			3	2	2	2	9	4	6	6
	T30_*Potamothrix moldaviensis* *	6	14				3	3	4	2	7
	Limnodrilus profundicola ****					2		1	1		
	Aulodrilus limnobius ****	2	4	1							
Naidinae	N6_*Piguetiella blanci* (1) *	1	4	1							2
	N7_*Vejdovskyella intermedia* (1) *						3	5			1

		Vengeron	Vengeron	Site 32	Site 53	Site 53	Site 78	Site 78	Site 6	Site 21	Site 36
		33 s	66 s	33 s	33 s	66 s	33 s	66 s	33 s	33 s	33 s
	N14_*Uncinais uncinata* (1) *										1
	Stylaria lacustris (1) ****			5							1
Lumbriculidae	LL3_*Stylodrilus heringianus* (1) *										1
	Stylodrilus lemani (1) ****								5		1
Haplotaxidae	H1_*Haplotaxis gordioides* (1) *									1	

Supplementary Table S5: number of specimens of each oligochaete species/lineage obtained per lake site with high-throughput DNA barcoding of 33 (33 s) and 66 specimens (66 s). The names of species followed by (1) correspond to sensitive species; * taxa identified using our local COI reference database; *** taxa identified by building a barcode tree; **** taxa identified by morphological analysis

		downstream WWTP (C. du Syndicat)	Source (Benenté)	source (Mentue)	source (Boiron)	Morges (Morges)	UNIL (Chamberonne)	Bourdigny (Avril)	Peney Bay (Rhône)	Divonne (Versoix)	downstream (Boiron)	Dardagny (Charmilles)	L'isle (Venoge)	Rte de Chancy (Merley)	Vengeron (Lake Geneva)	Site 32 (Lake Geneva)	Site 53 (Lake Geneva)	Site 78 (Lake Geneva)	Site 6 (Lake Geneva)	Site 21 (Lake Geneva)	Site 36 (Lake Geneva)
Morphology	Nb taxa	9	7	4	7	11	17	8	9	6	9	5	7	6	14	11	6	14	13	9	14
	% Tubificinae	51	5	83	0	92,8	14,7	99	90,2	89,8	87,5	98,1	11,6	98	91	84,8	100	91,8	95,1	95,8	86,4
	% Tubificinae without hair setae	32	3	0	0	91,8	9,8	55	63,7	15,3	53,8	72,8	11,6	7	46	48,5	33	23,5	35,9	47,2	35,9
	% Tubificinae with hair setae	19	2	83	0	1	4,9	44	26,5	74,5	33,7	25,2	0	91	45	36,4	67	68,4	59,2	48,6	50,5
	% Naidinae + Pristininae	48	87	0	0	3,1	45,1	0	0	10,2	0	1,94	0	0	6	15,2	0	7,1	0	0	3,9
	% Lumbriculidae	0	4	1	10,7	2,1	24,5	1	9,8	0	11,5	0	0	2	3	0	0	1	4,9	4,2	9,7
	% Enchytraeidae	1	4	16	85,7	0	14,7	0	0	0	0,96	0	88,4	0	0	0	0	0	0	0	0
Sequencing of 33 specimens	Nb taxa	6	9	3	7	9	11	5	11	3	10	9	12	2	11	11	5	9	11	11	14
	% Tubificinae	48,5	12,1	72,7	0	84,8	21,2	97	87,9	84,8	84,8	100	6,1	100	96,8	82,4	100	90,9	84,8	97	78,8
	% Tubificinae without hair setae	36,4	6,1	0	0	84,8	15,2	57,6	60,6	12,9	51,5	69,7	6,1	6	45,2	47,1	18,2	15,2	36,4	27,3	42,4
	% Tubificinae with hair setae	12,1	6,1	72,7	0	0	6,1	39,4	27,3	77,4	33,3	30,3	0	94	51,6	35,3	81,8	75,8	48,5	69,7	36,4
	% Naidinae + Pristininae	51,5	69,7	0	0	6,1	36,4	0	0	9,7	6,1	0	0	0	3,2	17,6	0	9,1	0	0	15,2
	% Lumbriculidae	0	3	3	12,5	6,1	30,3	3	12,1	0	9,1	0	0	0	0	0	0	0	15,2	0	6,1
	% Enchytraeidae	0	15,2	24,2	87,5	3	12,1	0	0	0	0	0	93,9	0	0	0	0	0	0	0	0
Sequencing of 66 specimens	Nb taxa	9	10					8	13	8	11	9		5	12		9	10			
	% Tubificinae	50	7,6					98,5	89,4	90,6	87,9	100		100	93,8		100	92,4			
	% Tubificinae without hair setae	34,8	3					62,1	59,1	17,2	50	69,7		8	51,6		25,8	19,7			
	% Tubificinae with hair setae	15,2	4,5					36,4	30,3	73,4	37,9	30,3		92	42,2		74,2	72,7			
	% Naidinae + Pristininae	50	78,8					0	0	9,4	3	0		0	6,3		0	7,6			
	% Lumbriculidae	0	1,5					1,5	10,6	0	9,1	0		0	0		0	0			
	% Enchytraeidae	0	12,1					0	0	0	0	0		0	0		0	0			

Supplementary Table S6: total number of taxa and percentages of oligochaete families/subfamilies obtained per site (streams and lake) with morphological analysis and high-throughput DNA barcoding of 33 and 66 specimens

Supplementary File S1 : COI sequences (313bp) corresponding to new lineages for the Swiss reference database (Vivien et al., 2017)

>Fridericia perrieri (source, Benenté)
CTAGCAAGTAACATAGCCCACGCTGGACCTTCAGTAGACTTAGCTATTTTTCTCTTCAC
CTAGCAGGTGCCTCCTCTATTCTAGGGGCAGTAAATTTCATTTCCACCGTAATTAACATA
CGATGACAAGGACTCCAATTAGAACGAATTCCCCTCTTTGTATGAGCAGTAACAATTACT
GTAGTCCTCCTACTGCTATCTCTTCCAGTTCTCGCCGGAGCAATTACAATACTTCTTACA
GATCGAAATCTCAATACATCATTTTTCGACCCAGCCGGGGGTGGAGACCCTATTTTATAT
CAACACTTATTT
>Enchytraeidae sp. (1) (source, Benenté)
CTAGCAAGAAATATAGCTCATGCCGGCCCTTCAGTAGACTTAGCCATTTTTTCACTTCAT
TTAGCCGGTGCCTCCTCCATTCTAGGAGCAGTAAACTTTATCTCAACCGTAATTAATATA
CGATGACAGGGACTTCAACTAGAACGAATTCCATTATTCGTCTGAGCAGTAACAATTACA
GTAGTTCTCCTTCTTCTCTCGCCTCCAGTACTTGCCGGAGCAATTACTATGCTACTCACC
GATCGCAACCTCAATACATCATTCTTTGACCCAGCAGGCGGGGGAGACCCCATTCTATAC
CAACACTTATTT
>Aulodrilus limnobius (source, Benenté)
CTAGCAAGTAATTTAGCACACTCAGGACCATCAGTAGACCTAGCAATTTTTTCGCTACAT
CTCGCCGGTGCTGCTTCAATTCTAGGTGCAATCAACTTTATTACAACAATAATTAACATA
CGATGAAAAGGGATACGACTAGAACGCATTCCACTATTCGTTTGAGCCGTAATTCTAACA
GTAATTCTCCTACTTTTAACACTCCCAGTGTTAGCAGGAGCCATCACTATACTTCTCACA
GACCGAAATCTAAACACTTCATTCTTCGACCCAGCAGGCGGGGGAGACCCAGTTCTATAC
CAACACTTATTC
>Enchytraeidae sp. (4) (source, Benenté)
CTATCTAGGAATCTAGCTCATGCAGGCCCATCTGTAGATCTAGCAATTTTTTCACTTCAT
TTAGCAGGTGCCTCATCTATTTTAGGAGCAGTAAATTTTATTACAACAGTTATTAATATA
CGATGACAAGGACTACGTCTAGAACGAATTCCCTTATCTGTATGAGCAGTAGTAATTACA
GTAGTACTACTCCTTCTTTCTCTTCCAGTTTTAGCTGGCGCCATCACCATATTATTAACA
GATCGAAACTTAAACACATCCTTTTTTGATCCAGCAGGAGGAGGAGACCCAATTCTCTAT
CAACACCTATTT
>Cernosvitoviella sp. (source, Mentue; source, Boiron)
CTAGCTAGAAATTTAGCACATGCCGGTCCCTCTGTAGACTTAGCCATTTTCTCACTTCAT
CTAGCTGGTGCCTCATCAATTTTAGGGGCAGTAAACTTCATTACTACAGTAATTAATATA
CGATGACAAGGACTACGCCTAGAACGAATCCCCCTATTTGTATGGGCCGTAGTAATTACA
GTAGTACTTCTATTATTATCCCTACCTGTACTTGCTGGAGCAATTACTATACTTCTAACC
GATCGTAACTTAAACACCTCATTTTTCGACCCCGCTGGAGGTGGTGACCCAATTTTATAT
CAACACCTTTTT
>Lumbriculidae g. sp. (source, Boiron)
CTGTCAAGAAATCTAGCACATGCAGGTCCTTCTGTAGACTTAGCAATTTTTTCTCTTCAT
TTAGCAGGAGCTTCATCAATCTTAGGTGCTGTAAATTTTATTACTACAGTAATTAATATA
CGATGAAATGGAATACACTTAGAACGAATTCCTCTATTTGTTTGATCAGTAATTATTACA
GTAGTACTACTTCTACTTTCAATACCTGTACTAGCGGGAGCTATTACAATATTATTAACA
GACCGTAATTTAAATACAGTTTTTTTTGATCCTGCAGGAGGAGGAGACCCTATTTTATAT
CAACACTTATTT
>Chamaedrilus chalupskyi (source, Boiron)
CTTTCACCCAACATAGCTCATGCTGGCCCATCAGTCGATCTTGCTATTTTCTCTTTACAT
TTAGCAGGAGCATCATCAATTCTAGGAGCAGTAAATTTTATTACAACTGTAATCAACATA
CGAATAAAAGGAATACAACTTGAACGAATACCACTATTTGTATGAGCTGTTGTAATTACA
GTTGTTCTTCTTCTTTTATCACTTCCAGTTCTTGCTGGAGCTATTACTATACTTCTAACA
GACCGAAATCTTAATACTTCATTCTTTGATCCTGCAGGAGGAGGTGACCCTATTCTTTAC
CAACACTTATTC
>Aulodrilus limnobius (Vengeron and site 32, Lake Geneva)
CTAGCCAGCAATCTAGCACATTCAGGACCATCAGTAGATCTAGCAATTTTCTCTCTACAT
CTTGCAGGTGCTGCCTCAATTCTAGGCGCAATCAACTTTATTACTACAATAATCAACATA
CGATGAAAAGGAATACGACTAGAACGAATTCCACTATTTGTATGAGCAGTAATCTTAACA
GTAATCCTACTACTCCTAACTCTACCAGTTCTAGCCGGTGCTATCACCATGCTACTAACA
GATCGAAATCTAAACACCTCATTCTTCGATCCTGCAGGAGGCGGGGACCCAGTTCTATAT
CAACATCTATTC
>Limnodrilus profundicola (site 53, 78, 6, Lake Geneva)

CTATCTAGTAATCTAGCTCACTCAGGACCATCTGTAGATCTTGCAATTTTCTCACTTCAT
TTAGCTGGTGTAGCATCAATTCTGGGGGCCATTAATTTCATTACAACAATGATTAATATA
CGGTGAAAAGGAATGCGGTTAGAACGAATTCCTTTATTTGTGTGATCAGTAATCATTACA
GTTGTCTTATTACTGTTAACCCTGCCTGTGCTAGCAGGAGCCATTACTATACTTTTAACA
GATCGAAATCTAAATACATCGTTCTTCGATCCTGCGGGGGGGGGAGATCCAGTACTGTAT
CAACACTTATTC
>Tubificinae sp. (with hair setae) (site 78, Lake Geneva)
CTTGCTGGCAATCTAGCCCACTCCGGCCCTTCAGTAGACCTAGCCATCTTTTCCCTTCAC
CTCGCTGGTGCCTCATCTATTTTAGGGGCAATTAATTTCATCACTACCATAATCAATATA
CGATGAAAAGGCATGCGCTTAGAACGCATCCCCCTATTTGTATGAGCAGTAATCATCACC
GTAGTTCTCCTTCTCCTTACACTTCCTGTTCTTGCTGGTGCTATTACAATACTCTTAACA
GACCGAAACTTAAACACTTCATTCTTCGATCCAGCCGGTGGGGGAGACCCTGTACTTTAC
CAACACTTATTC
>Enchytraeidae sp. (3) (source, Boiron)
CTCTCATCAAATATTGCTCATTCTGGACCATCTGTAGATTTAGCTATTTTTTCTCTCCAT
TTAGCTGGAGCTTCTTCTACTTTAGGAGCAATTAATTTTATTACAACAGTTATTAACATA
CGATGACAAGGTTTACGATTAGAACGAATTCCATTATTTGTTTGAGCTGTTACTATTACT
GTAGTCTTACTACTGCTATCTTTACCTGTCTTAGCTGGGGCTATTACTATATTATTAACA
GATCGAAATTTAAATACATCTTTTTTTGATCCTGCTGGTGGAGGGGATCCAATTTTATAT
CAACACTTATTT
>Stylaria lacustris (site 32, Lake Geneva)
CTATCTAGAAATTTAGCACACGCTGGTCCATCAGTAGATATGGCTATTTTTTCACTTCAT
TTAGCCGGTGCTTCTTCTATTTTAGGAGCAGTAAATTTTATTACAACAGTAATAAATATA
CGATGAAATGGGATACGTCTAGAACGAGTACCATTATTTGTATGAGCTGTAACACTTACA
GTAATTCTTCTTCTACTATCATTACCAGTACTAGCAGGAGCTATTACTATATTACTAACA
GATCGAAATCTAAATACATCATTCTTTGACCCAGCAGGAGGTGGAGACCCTATTTTATAT
CAACATTTATTT
>Quistadrilus multisetosus (site 53, Lake Geneva)
CTATCAGGAAATCTGGCACACTCCGGGCCTTCAGTAGACTTAGCCATCTTTTCCCTCCAC
TTAGCTGGTGCAGCATCAATCTTAGGGGCCATCAACTTCATCACCACAATAATTAACATA
CGGTGAAAAGGTATACGCCTAGAGCGTATTCCTCTATTTGTATGAGCTGTGATTCTAACA
GTAATTCTTCTTCTTCTAACACTACCAGTATTAGCTGGCGCAATCACTATACTTCTAACG
GACCGAAATCTAAATACCTCCTTCTTCGACCCGGCAGGAGGAGGGGACCCAGTTCTATAT
CAACACTTATTC
>Buchholzia appendiculata (UNIL, Chamberonne)
CTTGCAAGTAACATTGCTCACGCAGGACCTTCAGTAGACCTTGCTATTTTCTCTCTACAC
TTAGCAGGTGCATCATCTATTCTGGGCGCAGTAAATTTTATCTCAACAGTAGTAAATATA
CGTTGACAAGGCTTACAACTAGAACGAATCCCACTATTCGTTTGAGCTGTAACTATTACA
GTTGTTCTATTACTCCTATCTCTCCCAGTCCTTGCTGGAGCAATTACTATATTATTAACA
GATCGAAACTTAAATACATCATTTTTCGATCCAGCCGGGGGAGGTGACCCAATTCTATAT
CAACACTTATTC
>Henlea ventriculosa (UNIL, Chamberonne)
CTATCATCTAACATTGCTCATGCCGGGCCATCAGTAGACTTAGCAATTTTTTCTCTTCAC
TTAGCTGGTGCATCATCTATTTTAGGCGCAGTAAATTTCATTACTACAGTTATCAATATA
CGATGACAAGGTTTATCATTAGAACGAATTCCTTTATTTGTGTGAGCAGTTACAATCACT
GTAGTACTACTTCTCCTATCACTTCCTGTTCTTGCAGGAGCTATTACCATACTTCTAACA
GATCGTAATCTAAATACATCATTCTTCGATCCAGCGGGTGGGGGAGATCCAATTCTATAC
CAACACTTATTC
>Limnodrilus claparedianus cervix (Bourdigny, Avril)
CTAGCTAGAAACCTAGCACATTCTGGACCATCCGTAGATCTTGCAATTTTCTCTCTTCAC
TTAGCGGGGGTAGCATCTATTCTAGGTGCAATTAACTTCATTACCACAATAATTAATATA
CGCTGAAAAGGTATACGTCTAGAACGAATTCCATTATTTGTCTGATCTGTAATTATTACA
GTTATTCTTCTTCTCCTTACATTACCAGTTCTAGCAGGAGCAATTACCATGCTCCTAACA
GACCGAAATCTAAACACTTCATTCTTTGACCCTGCCGGGGGTGGAGACCCTGTACTATAT
CAACACTTATTT
>Stylodrilus lemani (site 6, 36, Lake Geneva)
TTAGCAAGAAATCTTGCACATAGCGGACCCTCAGTTGATTTAGCAATCTTTTCATTACAC
CTTGCTGGTGCCTCATCTATTTTGGGGGCCTTAAATTTTATTACCACAGTAGTAAATATG
CGGTGAAACGGACTACGATTAGAACGAATTCCTTTATTCGTCTGAGCTGTAACAATTACA
GTGGTTCTATTGCTTTTATCACTTCCTGTACTAGCAGGTGCGATTACTATACTTCTTACT

GACCGAAATTTAAATACTTCTTTTTTTGATCCTGCGGGGGGGGGTGATCCAGTCCTATAC
CAACACTTATTC
>Limnodrilus udekemianus (Morges, Morges; downstream, Boiron; Dardagny, Charmilles)
TTAGCAGGGAATCTCGCTCATTCCGGACCTTCTGTGGACCTTGCCATTTTTTCTCTTCAT
TTAGCCGGAATCTCCTCAATTCTAGGGGCTATTAACTTCATTACCACTATAATTAATATA
CGATGAAAAGGAATACGACTTGAACGAATTCCTTTATTTGTGTGATCTGTGATTATTACT
GTAGTCTTACTTCTATTAACTCTCCCTGTACTAGCAGGAGCAATCACAATACTCCTAACA
GATCGAAATCTAAATACATCATTTTTCGACCCGGCTGGGGGAGGTGATCCTGTCCTATAT
CAACACTTATTC
>Rhyacodrilus falciformis (L’isle, Venoge)
TTAGCTAGAAATATAGCCCATGCTGGACCATCAGTAGATATAGCAATTTTTTCTTTACAT
TTAGCGGGTGCCTCATCAATTCTTGGTGCAGTAAATTTTATTACAACTGTAATAAATATA
CGATGAACAGGAATAAAACTAGACCGAATTCCATTATTTGTATGGGCTGTTATATTAACA
GTGATTTTACTTTTACTATCACTTCCCGTTTTAGCTGGGGCTATTACAATGTTACTAACA
GATCGTAATCTAAATACTTCTTTTTTTGATCCTGCTGGTGGTGGAGATCCTATTTTATAT
CAACATCTATTT
>Enchytraeidae sp. (6, 27) (L’isle, Venoge)
CTTGCAAGAAACATAGCCCATGCCGGTCCATCAGTAGATCTAGCAATTTTTTCACTACAT
TTAGCAGGTGCATCATCAATTTTAGGAGCAGTAAACTTTATCTCAACAGTAATTAATATA
CGATGACAAGGCCTACAACTGGAACGAATCCCCCTATTTGTATGAGCTGTTACAATTACA
GTTGTCCTTCTCCTACTATCACTCCCAGTACTAGCAGGTGCAATCACCATACTACTAACA
GATCGAAATTTAAACACATCCTTCTTTGATCCTGCAGGTGGAGGGGATCCAATTCTATAT
CAACATCTATTC
>Enchytraeidae sp. (17, 25) (L’isle, Venoge)
CTAGCAAGAAATATTGCCCATGCCGGCCCATCTGTAGACTTAGCTATCTTTTCACTACAC
TTAGCAGGAGCTTCTTCCATTTTAGGAGCTGTAAACTTTATTACGACTGTGATAAACATA
CGCAGACAAGGATTAACTCTAGAGCGAATTCCATTATTTGTGTGAGCCCTAGTTATCACA
ACAACAATACTACTTTTAGCCCTCCCGGTTTTAGCAGGAGCAATTACCATACTTTTAACC
GATCGAAACTTAAACACTTCATTCTTTGACCCTGCAGGAGGGGGTGACCCAATCCTATAT
CAACACTTATTT
>Enchytraeidae sp. (20, 21) (L’isle, Venoge)
-TTTCATCAAATATTGCTCATTCTGGTCCTTCAGTAGATTTAGCTATTTTCTCCTTACAC
TTAGCAGGGGCCTCCTCCATCTTAGGGGCCATTAATTTCATTACTACTGTTATTAACATA
CGCTGACAAGGGCTTCAACTAGAACGAATTCCCCTTTTTGTTTGAGCCGTATCAATTACA
GTAGTTCTACTCTTACTCTCTCTTCCAGTTTTAGCAGGAGCAATTACTATACTTCTAACA
GATCGAAATTTAAATACCTCATTCTTTGACCCAGCCGGGGGGGGAGACCCAATTCTCTAC
CAACACCTATTC
>Enchytraeidae sp. (32) (L’isle, Venoge)
CTAGCAAGGAATATAGCACATGCAGGACCCTCTGTAGATTTAGCCATTTTTTCTCTTCAT
TTAGCAGGAGCTTCTTCCATTTTAGGCGCAGTCAACTTCATCTCCACTGTAATTAATATA
CGATGACAAGGTCTTCAACTAGAACGAATTCCATTATTTGTTTGAGCTGTGACCATTACA
GTAGTGCTATTACTTCTTTCTTTACCAGTACTTGCTGGGGCAATTACTATACTTTTAACA
GATCGAAATTTAAATACATCATTCTTTGACCCAGCAGGAGGCGGAGACCCTATCCTATAT
CAACACTTGTTT
>Tubificinae sp. (with hair setae) (Dardagny, Charmilles)
CTAGCTAGAAACTTAGCACACTCTGGACCATCTGTAGACTTAGCTATCTTCTCACTACAT
TTAGCTGGTGTTGCATCAATCCTAGGAGCTATTAATTTCATTACTACAATAATTAACATG
CGTTGAAAAGGTATACGCCTAGAACGAATTCCATTATTCGTTTGATCAGTAATCATTACT
GTAGTCCTTCTATTACTTACACTACCAGTACTAGCCGGCGCAATTACTATACTTCTTACA
GACCGAAATCTAAATACCTCATTCTTTGATCCGGCTGGGGGTGGTGATCCTGTCCTCTAC
CAACATCTATTC
